# Case of Congenital Cataract; Total Blindness for Sixteen Years; Operation, and Subsequent Enjoyment of Sight

**Published:** 1859-01

**Authors:** John S. Rohrer

**Affiliations:** Philadelphia


ART. III.—*Case of Congenital Cataract; Total Blindness for Sixteen Years; Operation on Both Eyes; Keratonyxis and Sclerotonyxis; Restoration to the Enjoyment of Sight; Subsequent Unimpaired Vision up to the Present Time, a Period of Twenty-one Years.* By JOHN S. ROHRER, M.D., of Philadelphia.

THE subject of this case, at the time of the operation, was a young lady sixteen years of age, who had been afflicted with cataract from her birth.

Her history in connection with this affection has been traced back thirty-seven years, and, therefore, contains some points of interest.

When the patient was about eight years old, her eyes were examined with a view to an operation, but I have not been able to learn what circumstances were present at the time to render the result doubtful; still it is evident that some complication with the cataract, either local or general, must have existed, for, upon further consideration, the original design of an operation was relinquished. Whatever the delaying cause may have been, it is certain that many oculists deem it proper to wait for an "age when reason and reflection in the patients will enable them more properly to estimate the value of sight, and render them more sensible of the propriety of submitting patiently to the operation."

Her parents, having been informed that the prognosis was unfavorable, did not think it necessary to seek further medical advice, and permitted her thus to grow up to the age of womanhood, regardless of the propriety of sending her to any institution of learning for this class of pupils. Being thus deprived of sight and the benefits of education, and living in a secluded situation with her parents, who were now advanced in years, and moreover being naturally intelligent, and of a sprightly disposition, she sought her friends and relatives at a distance, to aid her in one more effort to obtain her sight; and in the confident expectation of this favor she was not disappointed. Her friends at once encouraged her in the enterprise; and in view of her helpless situation offered her every facility in obtaining medical aid, which finally resulted in submitting herself to my care, with a firm resolution on her part to bear the operation with all the strength and fortitude in her power. All the advantages and disadvantages of a successful or unsuccessful operation were fairly laid before her, and having arrived at an age of discretion, she was advised to deliberate as to the course she intended to adopt, and in either case to abide by the consequences.

The eyes were deeply sunk in the orbits, and rather smaller than natural, and the cataracts presented a dense appearance of a light-gray color, but were not as large as cataracts of a soft and caseous consistence are usually described.

Having duly considered the nature of the case, I proposed performing the anterior operation, or Keratonyxis, and for this purpose provided myself with several very slender needles, of different shapes and sizes, to meet any emergency. The one I proposed to employ was expressly made by the late John Rorer, cutler, of this city, and was expected to pass through the cornea with great facility. It was a slender, straight needle, with a double edge for a short distance, slightly spear-pointed, and not much larger than a common sewing needle, but possessing sufficient strength to pass through the dense cornea without the risk of breaking.

The only impediments visible to a successful operation were the deep-set eyes already mentioned, a slight tendency to ophthalmia, and a chronic inflammatory affection of the eyelids, to which she was subject, and a restless and rolling motion of the eyes which she had acquired, and which is so common in persons born blind, impeding the easy introduction of the needle.

Having previously prepared the system of the patient, the extract of belladonna, diluted with water to the consistence of cream, was smeared on the brows and eyelids. After the pupils were sufficiently dilated the patient was seated on a chair before me, near the light of a window, the right eyelid being secured by two fingers of an assistant, who at the same time supported the head.

The needle was introduced through the cornea, about a line or more in advance of its junction with the sclerotica, and passed directly through the pupil to the capsule, which was lacerated by a few strokes from above downward, and then passed into the substance of the lens, which latter was found to be soft and pulpy; a few motions of the needle completed the operation, and it was gently withdrawn, leaving the capsule and lens in situ.

The difficulty experienced in this operation was the resistance of the cornea to the needle. It required much more force to push it through the cornea than was anticipated, the latter yielding to indentation before the needle could be passed through it, which caused the iris to contract, so that it became necessary to arrest the motion of the instrument for a few seconds to permit the pupil to dilate sufficiently to finish the operation. In consequence, therefore, of the embarrassment experienced by the tardy passage of the needle through the cornea, I resolved, in the left eye, to operate through the sclerotica behind the iris.

As soon as the patient was composed I introduced Scarpa's needle, made straight and reduced to the smallest possible size, into the left eye through the sclerotica, about a line and a half behind the margin of the cornea, and a little above the centre of the ball, and passed it to the capsule, which I lacerated by drawing it across in several directions, when it was pushed into the substance of the lens, which was cautiously broken up as in the other eye, and left remain for absorption. Sight was also observed in this eye as soon as the needle was withdrawn. The extract of belladonna was now smeared round the margin of the orbit, cool dressings were applied, and the patient was removed into a dark room prepared for her. On the second day, when the dressings were temporarily removed, the patient could discern light from the window as before. No inflammation of any account followed, and in the course of a week the cataracts were sufficiently absorbed to enable the patient to see objects in the room. The process of absorption went on gradually, and the sight continued to improve, so that in a few weeks more the patient could walk from one room to another and discern persons and things around her. But in the exercise of this new faculty she had many novel difficulties to encounter. As to distance she had no appreciable knowledge by sight, and when left to take care of herself would frequently walk up rather close to objects, and sometimes bring her head too suddenly in contact with the wall, or step over articles of furniture near her. It required, therefore, some days training to get over these difficulties and to judge correctly of distance.

What is equally remarkable in sudden recovery of sight from congenital cataract, as exemplified in this case, is the illusion experienced at first in handling objects. For instance, the patient in reaching for a knife or fork would heedlessly, as it were, direct the hand to the wrong end, and take hold of the blade instead of the handle. The knowledge, it must be understood, of directing the hands properly is acquired by habit, as in the case of little children.

With colors she was both gratified and amused, as presenting the greatest novelty in her late acquisition of sight; and the impression which they made on her mind was such that the name of any color once mentioned was never forgotten, or one mistaken for another.

Several incidents may be related to show the force of habit, and the training of the eye which is necessary to enable the patient to distinguish objects rightly, and appreciate the use of colors and their combinations in representing objects.

Colored pictures representing dogs, cats, cows, horses, etc., which were presented to her soon after recovery of vision, could not be recognized by her as such, although they were already familiar to her sight; and when directed to examine them closely, could only see black, white, or yellow spots, and projections in different directions, according to the nature of the painting; but by a little practice, after being informed that the colors and curves represented the shape or figure of the animals, and by tracing the outlines with the finger, and making the same curves with the hand over the body of the animals in question, she soon comprehended the representation. The deception to her inexperienced eyes, it may easily be imagined, was on account of the flatness of the surface of the paper or canvas on which the animals were painted, having been accustomed to know them only by their bulk in feeling and handling them; each color, shade, or line, being recognized by her as distinct objects.

From the above will be seen the difficulty she had experienced in recognizing the resemblance or representation of things with which she had already been familiar by sight; but many objects were brought before her to test her vision which had never been seen by her, and which were only known to her by the sense of touch; such of course she could not recognize by sight, either by their actual presence or representation on paper, however well executed, thus causing doubts among some of her incredulous friends as to the integrity of this newly acquired faculty. But their delusions were soon dispelled; a little familiarity with persons and things soon enabled her to recognize every object near her, and to discern the features of different persons in her presence, until finally, by the aid of glasses and time, she became familiar with smaller objects, and could distinguish letters of ordinary sized type. It may be proper here to state that my personal observations ceased soon after the restoration of her sight, my patient having removed with her friends to the "West," and for the last fifteen years I have only occasionally heard from her; but the accounts which I received were very flattering; her sight continued to improve for several years subsequent to the operation, and that restless, rolling motion of the eyes, at first so troublesome, had almost entirely disappeared. A few years ago I received a communication from her giving a short account of her sight, with a request to procure cataract glasses. At this time she could see very well without them, and had not used any for several years.

